# Interactive associations of smoking and physical activity with metabolic syndrome in adult men in Korea

**DOI:** 10.3389/fpubh.2023.1281530

**Published:** 2023-11-16

**Authors:** Minjun Kim, Joonwoong Kim, Inhwan Lee

**Affiliations:** ^1^Department of Physical Education, Yongin University, Yongin, Republic of Korea; ^2^Department of Convergence, Seowon University, Cheongju, Republic of Korea; ^3^Department of Anti-aging Healthcare, Changwon National University, Changwon, Republic of Korea; ^4^Department of Human Senior Ecology Cooperative Course, Changwon National University, Changwon, Republic of Korea

**Keywords:** smoking status, physical activity, metabolic syndrome, interactive association, Korean men

## Abstract

**Introduction:**

This study aimed to investigate the association of smoking and physical activity (PA) with metabolic syndrome (MetS) in adult men in Korea.

**Methods:**

This study analyzed data of 7,229 adult men aged 19–64 years obtained from the 2014–2021 Korea National Health and Nutrition Examination Survey (KNHANES). Information on smoking habits was obtained using KNHANES data, while that on total PA (TPA), leisure-time PA (LTPA), and occupational PA (OPA) was collected using the Global Physical Activity Questionnaire. Smoking status was classified into non-smokers and smokers, and PA was categorized into three groups (total, leisure time, and occupational) according to the time spent engaging in moderate or high-intensity PA areas. The diagnosis of MetS was based on the Adult Treatment Program III of the National Cholesterol Education Program and Koreans’ waist circumference criteria.

**Results:**

Logistic regression revealed that the risk of MetS was significantly lower in non-smokers than in smokers, even after adjusting for all covariates. The risk of MetS was significantly lower in individuals who engaged in at least 150 min of moderate- and high-intensity TPA or LTPA per week than in those who did not engage in PA. Furthermore, smokers who engaged in at least 150 min of moderate- to high-intensity TPA and LTPA per week had a significantly lower risk of MetS than those who did not engage in PA. Meanwhile, OPA was not associated with MetS.

**Conclusion:**

The findings suggest that engaging in moderate- to high-intensity TPA or LTPA for at least 150 min per week attenuates the risk of MetS caused by smoking.

## 1. Introduction

Metabolic syndrome (MetS) is defined as the clustering of abnormalities in three or more of the following five components: waist circumference (WC), blood pressure (BP), fasting blood glucose (FBG), high-density lipoprotein cholesterol (HDL-C), and triglyceride (TG) (1). Individuals with MetS may experience symptoms depending on the degree of deviation in their levels of these components; however, most people with MetS remain asymptomatic, making early diagnosis difficult (2). MetS, if left untreated, may lead to cardiovascular disease (3–5) and metabolic disorders, such as type 2 diabetes mellitus (3, 6, 7), as well as other serious health conditions (8–11). Therefore, modifiable risk factors should be identified to prevent MetS.

Smoking is among the most well-established risk factors for MetS (12). Although the exact pathophysiological role of smoking in MetS remains unclear, it is postulated that smoking causes MetS by reducing peripheral insulin sensitivity (13, 14), altering lipoprotein metabolism (15, 16), and damaging the vascular endothelium, resulting in endothelial dysfunction (17). In fact, many epidemiological studies have shown that smoking is significantly associated with decreased HDL-C (15, 16, 18), increased WC (18), elevated TG (19), increased FBG (20), and increased resting BP (21) and that smokers have a higher risk of MetS than non-smokers (22–25).

In contrast, regular physical activity (PA) contributes to the prevention and alleviation of MetS. PA can be classified into various domains and types (occupational/leisure); although the optimal PA for preventing MetS remains unknown, increasing evidence suggests that regular moderate- or high-intensity PA of at least 150 min per week, as recommended by the World Health Organization (WHO), helps prevent and alleviate MetS (26–29).

Although previous research has extensively investigated the individual impact of smoking and PA on MetS, the exact effect of the interplay between them remains unknown. Some studies showed that the protective effects of PA against MetS are also evident among smokers. For example, a cross-sectional study on workers in Taiwan reported that the risk for low HDL-C and high TG decreased with the increase in the duration of PA, irrespective of the smoking status (30). Furthermore, an exercise interventional study conducted on male college students in Korea showed that the WC declined significantly with participation in PA, even in smokers (31). However, these studies investigated a particular subset of the population and did not provide quantitative evidence supporting the effect of PA on the prevention and alleviation of MetS among smokers. Using large samples to evaluate the association between smoking and PA and MetS, considering different domains and durations of PA, may provide broader and more detailed information about PA that may be useful in preventing MetS in smokers. The smoking rate among Korean men is higher than that among women, and a marked difference exists between the self-reported and actual smoking rates among women (32). Therefore, this study aimed to investigate the association of smoking and PA with MetS in adult men in Korea.

## 2. Materials and methods

### 2.1. Data source

This study used data from the nationally representative Korea National Health and Nutrition Examination Survey (KNHANES; 2014–2021), which is conducted annually to assess Koreans’ health and nutrition and provide data for policy making. The KNHANES was conducted after obtaining approval from the Institutional Review Board at the Korea Disease Control and Prevention Agency and written consent from all participants. KNHANES uses Korea’s population and housing census and resident registration population as a data extraction framework to ensure the representativeness of the non-institutionalized Korean population. After selecting the sample using the stratified cluster sampling design method, all individuals aged 1 year or older within the sample household is selected. The household members are selected as participants in the survey. The design and sampling of the survey have been previously described in detail (33).

**Figure 1:**
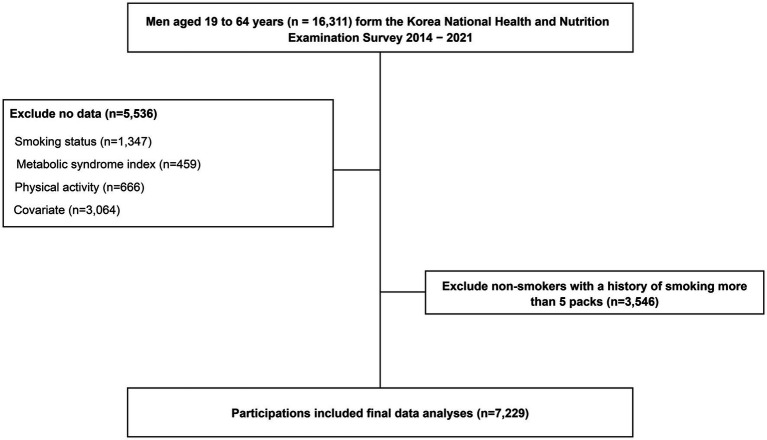
Flow chart of eligible participants in the study.

Adult men aged 19–64 years who participated in the KNHANES from 2014 to 2021 were considered for this study (*n* = 16,311). This study excluded participants with missing data on smoking (*n* = 1,347), MetS index (*n* = 459), PA (*n* = 666), and covariates (*n* = 3,064) and those who currently do not smoke cigarettes or e-cigarettes but have smoked more than 100 cigarettes in their lifetime, as it was impossible to control for the effect of the past history of smoking on MetS (*n* = 3,546). Thus, 7,229 participants were included in the analysis (Figure 1).

### 2.2. Assessment of anthropometric data and definition of metabolic syndrome

Height and body weight were measured using an automatic stadiometer (seca 274, seca, Germany) and scale (GL-6000-20, Gtech, Korea), respectively. The body mass index (BMI) was calculated by dividing weight (kg) by the square of the height (m^2^). WC was measured using an anthropometric measuring tape at the midpoint between the lowest rib and the top of the iliac crest in a standing position. BP was measured using a mercury-free BP cuff (Greenlight300, Accoson, UK) after having the participant sit down for at least 10 min. Three BP measurements were performed, and the average value was used. Blood samples were obtained from the brachial vein of the non-dominant arm after 8–12 h of fasting. The enzymatic method was employed to measure TG and HDL-C, and the hexokinase method was employed to analyze FBG using an analyzer (Labospect 008AS, Hitachi, Japan).

The diagnosis of MetS was made if three of the following criteria were met, with reference to the Adult Treatment Program III of the National Cholesterol Education Program (34) and WC criteria for Koreans (35): (1) WC ≥ 90 cm; (2) ≥130/85 mmHg or use of antihypertensive medications; (3) TG ≥150 md/dl or use of lipid-lowering medications; (4) HDL-C < 40 mg/dl; and (5) FBG ≥ 100 mg/dl or use of anti-hypoglycemic medications.

### 2.3. Smoking status

Smokers were defined as participants who answered “smoke every day” or “sometimes” to the question “Do you currently smoke regular cigarettes or electronic cigarettes?” Non-smokers were defined as individuals who had never smoked electronic cigarettes among those who answered “never smoked” or “less than 100 cigarettes” to the question “How many regular cigarettes have you smoked in your lifetime?”

### 2.4. Physical activity

The duration of moderate- and high-intensity leisure-time PA (LTPA), occupational PA (OPA), and total PA (TPA; LTPA + OPA) per week was calculated using a Korean version of the Global Physical Activity Questionnaire (36). The duration of PA was categorized based on the domain (occupational/leisure) of PA according to the WHO guidelines (37) for adults as follows: (1) inactive: 0 min, (2) insufficiently active: 1–149 min, and (3) active: ≥150 min.

### 2.5. Covariates

The covariates in this study were based on the contents used in the KNHANES self-administered questionnaire. These covariates were defined as a motive for previous studies that analyzed the relationship between smoking and MetS using KNHANES data (38). This study’s covariates included demographic factors (age, education level, and marital status), socioeconomic status (SES; monthly household income, employment status, and residential region), and health-related parameters (weekly alcohol consumption, perceived stress, BMI, and daily energy intake).

### 2.6. Statistical analysis

In this study, continuous variables were presented as means and standard deviations, and categorical variables were presented as percentages. The participants’ characteristics according to smoking status were compared using the independent t-test and chi-square test, and their characteristics according to PA were compared using linear trends in the one-way analysis of variance and linear-by-linear association in the chi-square test. Binomial logistic regression was performed to analyze the associations of smoking and PA (by domain) with MetS, and the results were presented as odds ratios (ORs) and 95% confidence interval. All statistical analyses were performed using the IBM SPSS Statistics for Windows, version 28.0 (IBM Corp, Armonk, NY, USA). Statistical significance was set at *p* < 0.05.

## 3. Results

### 3.1. Participants’ characteristics according to smoking status

Table 1 shows the descriptive statistics for the participants’ characteristics according to smoking status. MetS, high WC, elevated systolic BP (SBP)/diastolic BP (DBP), high TG, low HDL-C, and high FBG were more prevalent in smokers than in non-smokers (*p* < 0.001). Smokers were older (*p* < 0.001), had a lower monthly household income (*p* < 0.001), were more likely to be married (*p* < 0.001), were more likely to be employed (*p* < 0.001), had lower education (*p* < 0.001), and were less likely to live in an urban region (*p* < 0.001) than non-smokers. Furthermore, the proportion of alcohol consumption at least two times a week (*p* < 0.001) and high perceived stress (*p* < 0.001) was higher among smokers than among non-smokers. Smokers also had a longer weekly OPA (*p* < 0.001) and greater daily energy intake (*p* < 0.001) but lower weekly LTPA (*p* < 0.001) than non-smokers. The components of the MetS index, namely, WC (*p* < 0.001), SBP (*p* < 0.001), DBP (*p* < 0.001), TG (*p* < 0.001), and FBG (*p* < 0.001) were higher, and the HDL-C was lower (*p* < 0.001) in smokers than in non-smokers.

**Table 1:** Description of measured parameters according to smoking status.

Variable	Total (*n* = 7,229)	Smoking status		
		Non-smokers (*n* = 2,995/43.3%)	Smokers (*n* = 4,234/56.7%)	*p-*value
Demographic factors				
Age (years)	41.1 ± 13.0	38.0 ± 13.2	43.3 ± 12.3	<0.001
Education, *n* (%)				<0.001
Lower than high school	809 (8.8)	200 (5.2)	609 (11.6)	
High school	2,969 (43.0)	1,156 (38.6)	1,813 (44.8)	
College	3,451 (48.2)	1,639 (54.2)	1,812 (43.6)	
Marital status, *n* (%)				<0.001
Married	4,257 (54.2)	1,592 (47.7)	2,665 (59.2)	
Widowed/divorced	352 (3.9)	53 (1.4)	299 (5.9)	
Unmarried	2,620 (41.9)	1,350 (50.9)	1,270 (34.9)	
Socio-economic status				
Household income (10,000 won/month)	487.6 ± 314.2	523.0 ± 323.1	462.6 ± 305.3	<0.001
Employment status, *n* (%)				<0.001
Employed	5,775 (78.8)	2,289 (74.2)	3,486 (78.8)	
Unemployed	1,454 (21.2)	706 (25.8)	748 (21.2)	
Region, *n* (%)				<0.001
Urban	6,010 (85.9)	2,568 (88.1)	3,442 (84.2)	
Rural	1,219 (14.1)	427 (11.9)	792 (15.8)	
Health-related parameters				
Alcohol consumption, *n* (%)				<0.001
≥2	2,479 (32.4)	576 (17.8)	1,903 (43.7)	
<2	4,750 (67.6)	2,419 (82.2)	2,331 (56.3)	
Perceived stress level, *n* (%)				<0.001
Low	5,067 (70.0)	2,262 (75.4)	2,805 (65.9)	
High	2,162 (30.0)	733 (24.6)	1,429 (34.1)	
Physical activity				
TPA (min/week)	153.8 ± 354.8	151.1 ± 302.7	155.8 ± 387.4	0.569
LTPA (min/week)	83.6 ± 161.8	99.6 ± 167.4	72.3 ± 156.8	0.004
OPA (min/week)	70.2 ± 307.9	51.5 ± 240.3	83.5 ± 347.3	<0.001
BMI (kg/m^2^)	24.6 ± 3.6	24.6 ± 3.6	24.7 ± 3.6	0.293
Energy intake (kcal/day)	2447.7 ± 1041.6	2377.0 ± 954.4	2497.8 ± 1096.4	<0.001
Metabolic syndrome index				
MetS prevalence, *n* (%)	2,188 (28.1)	704 (21.6)	1,484 (33.1)	<0.001
High WC, *n* (%)	2,389 (33.0)	892 (29.8)	1,497 (35.4)	<0.001
High SBP/DBP, *n* (%)	2,640 (36.5)	938 (31.3)	1,702 (40.2)	<0.001
High TG, *n* (%)	3,147 (43.5)	954 (31.9)	2,193 (51.8)	<0.001
Low HDL-C, *n* (%)	1,760 (24.3)	599 (20.0)	1,161 (27.4)	<0.001
High FBG, *n* (%)	2,593 (35.9)	1,739 (41.1)	854 (28.5)	<0.001
WC (cm)	86.3 ± 9.6	85.6 ± 9.8	86.8 ± 9.5	<0.001
SBP (mmHg)	118.6 ± 13.9	117.6 ± 12.9	119.2 ± 14.5	<0.001
DBP (mmHg)	78.5 ± 10.1	77.8 ± 9.7	79.0 ± 10.3	<0.001
TG (mg/dl)	165.0 ± 143.5	136.2 ± 114.7	185.5 ± 157.7	<0.001
HDL-C (mg/dl)	47.6 ± 11.3	48.6 ± 10.9	47.0 ± 11.5	<0.001
FBG (mg/dl)	101.1 ± 25.0	98.3 ± 22.2	103.1 ± 26.7	<0.001

TPA, total physical activity; LTPA, leisure time physical activity; OPA, occupational physical activity; BMI, body mass index; MetS, metabolic syndrome; WC, waist circumference; SBP, systolic blood pressure; DBP, diastolic blood pressure; TG, triglyceride; HDL-C, high-density lipoprotein cholesterol; FBG, fasting blood glucose.

### 3.2. Participants’ characteristics according to PA

Table 2 shows the descriptive statistics for the participants’ characteristics according to PA (by weekly duration of TPA). First, the prevalence of MetS and the proportion of participants with high WC, high SBP/DBP, high TG, low HDL-C, and high FBG showed a significant linear trend according to the level of TPA (*p* < 0.001). A negative linear association was observed between PA and age (*p* < 0.001), marital status (*p* < 0.001), smoking (*p* < 0.001), alcohol consumption (*p* < 0.001), WC (*p* = 0.002), SBP (*p* < 0.001), DBP (*p* < 0.001), TG (*p* < 0.001), FBG (*p* < 0.001), and MetS prevalence (*p* < 0.001). Moreover, a positive linear association was observed between PA and BMI (*p* = 0.001), monthly household income (*p* < 0.001), education (*p* < 0.001), and residential region (*p* = 0.001). Individuals with a high PA level were younger, single, consumed less alcohol, did not smoke, and had lower WC, SBP, DBP, TG, and FBG levels and MetS prevalence than those with a low PA level. Furthermore, individuals with a high PA level had higher BMI, higher monthly household income, were more educated, and were likelier to live in an urban region than those with a low PA level.

**Table 2:** Description of parameters according to the total physical activity level.

Variable	Total physical activity level			
	Inactive (*n* = 3,960/54.8%)	Insufficiently active (*n* = 1,257/17.4%)	Active (*n* = 2,012/27.8%)	*p* for linear trend
Physical activity				
TPA (min/week)	0.0 ± 0.0	79.0 ± 35.9	503.4 ± 528.4	<0.001
LTPA (min/week)	0.0 ± 0.0	67.2 ± 42.7	258.4 ± 220.2	<0.001
OPA (min/week)	0.0 ± 0.0	11.8 ± 30.8	245.0 ± 545.7	<0.001
Demographic factors				
Age (years)	43.1 ± 13.0	40.4 ± 12.1	37.7 ± 12.7	<0.001
Education, *n* (%)				<0.001
Lower than high school	617 (15.6)	81 (6.4)	111 (5.5)	
High school	1,682 (42.5)	413 (32.9)	874 (43.4)	
College	1,661 (41.9)	763 (60.7)	1,027 (51.1)	
Marital status, *n* (%)				<0.001
Married	2,434 (61.5)	760 (60.5)	1,063 (52.8)	
Widowed/divorced	244 (6.2)	44 (3.5)	64 (3.2)	
Unmarried	1,282 (32.3)	453 (36.0)	885 (44.0)	
Socio-economic status				
Household income (10,000 won/month)	453.2 ± 306.1	545.9 ± 327.9	519.1 ± 312.7	<0.001
Employment status, *n* (%)				0.324
Employed	3,120 (78.8)	1,062 (84.5)	1,593 (79.2)	
Unemployed	840 (21.2)	195 (15.5)	419 (20.8)	
Region, *n* (%)				0.001
Urban	3,231 (81.6)	1,078 (85.8)	1,701 (84.5)	
Rural	729 (18.4)	179 (14.2)	311 (15.5)	
Health-related parameters				
Smoking, *n* (%)	2,485 (62.8)	673 (53.5)	1,076 (53.5)	<0.001
Alcohol consumption, *n* (%)				<0.001
≥2	1,451 (36.6)	421 (33.5)	607 (30.2)	
<2	2,509 (63.4)	836 (66.5)	1,405 (69.8)	
Perceived stress level, *n* (%)				0.144
Low	2,767 (69.9)	849 (67.5)	1,451 (72.1)	
High	1,193 (30.1)	408 (32.5)	561 (27.9)	
BMI (kg/m^2^)	24.5 ± 3.7	24.7 ± 3.5	24.9 ± 3.5	0.001
Energy intake (kcal/day)	2401.1 ± 1010.4	2465.7 ± 1014.5	2528.2 ± 1111.7	<0.001
Metabolic syndrome index				
MetS prevalence, *n* (%)	1,368 (34.5)	373 (29.7)	447 (20.4)	<0.001
High WC, *n* (%)	1,389 (35.1)	391 (31.1)	609 (30.3)	<0.001
High SBP/DBP, *n* (%)	1,575 (39.8)	439 (34.9)	626 (31.1)	<0.001
High TG, *n* (%)	1,864 (47.1)	536 (42.6)	747 (37.1)	<0.001
Low HDL-C, *n* (%)	1,559 (39.4)	458 (36.4)	576 (28.6)	<0.001
High FBG, *n* (%)	1,074 (27.1)	299 (23.8)	387 (19.2)	<0.001
WC (cm)	86.6 ± 10.0	86.2 ± 9.1	85.8 ± 9.2	0.002
SBP (mmHg)	119.2 ± 14.3	117.9 ± 13.5	117.8 ± 13.2	<0.001
DBP (mmHg)	79.0 ± 10.2	78.4 ± 10.1	77.4 ± 9.7	<0.001
TG (mg/dl)	174.9 ± 155.3	162.7 ± 123.9	147.1 ± 128.3	<0.001
HDL-C (mg/dl)	46.8 ± 11.3	47.8 ± 11.1	49.2 ± 11.2	<0.001
FBG (mg/dl)	103.2 ± 27.6	99.8 ± 20.9	97.8 ± 21.6	<0.001

TPA, total physical activity; LTPA, leisure time physical activity; OPA, occupational physical activity; BMI, body mass index; WC, waist circumference; SBP, systolic blood pressure; DBP, diastolic blood pressure; TG, triglyceride; HDL-C, high-density lipoprotein cholesterol; FBG, fasting blood glucose.

### 3.3. Binomial logistic regression for the association between smoking and MetS

Table 3 shows the OR estimates for MetS according to smoking status. The OR for MetS (OR = 0.556, *p* < 0.001) in non-smokers was significantly lower than that in smokers (OR = 1). The results retained significance in model 2, adjusted for age (OR = 0.679, *p* < 0.001), and model 3, adjusted for age, SES, and health-related factors (OR = 0.751, *p* < 0.001).

**Table 3:** OR and 95% CI of metabolic syndrome according to smoking status.

Variable	Model 1		Model 2		Model 3	
	OR (95% CI)	*p*-value	OR (95% CI)	*p*-value	OR (95% CI)	*p*-value
Smokers	1 (reference)		1 (reference)		1 (reference)	
Non-smokers	0.556 (0.494–0.627)	<0.001	0.679 (0.599–0.770)	<0.001	0.751 (0.669–0.843)	<0.001

OR, odds ratio; CI, confidence interval. Model 1: Unadjusted. Model 2: Adjusted for age. Model 3: Model 2 + education, income, marital status, employment status, region, drink, perceived stress level, total physical activity time, energy intake.

### 3.4. Binomial logistic regression for the association between PA and MetS

Table 4 shows the OR estimates for MetS according to the duration of PA by domain. First, regarding the TPA, the insufficiently active (OR = 0.816, *p* = 0.012) and active (OR = 0.522, *p* < 0.001) groups had a significantly lower OR for MetS than the inactive group (OR = 1). However, in the age-adjusted model (model 2; insufficiently active, OR = 0.911, *p* = 0.265; active, OR = 0.651, *p* < 0.001) and age-, SES-, and health-adjusted model (model 3; insufficiently active, OR = 0.912, *p* = 0.214; active, OR = 0.685, *p* < 0.001), only the active group had a significantly lower OR for MetS, with no significant results for the insufficiently active group.

**Table 4:** OR and 95% CI of metabolic syndrome according to the physical activity level stratified by type.

Variable	Model 1		Model 2		Model 3	
	OR (95% CI)	*p*-value	OR (95% CI)	*p*-value	OR (95% CI)	*p*-value
TPA						
Inactive	1 (reference)		1 (reference)		1 (reference)	
Insufficiently active	0.816 (0.697–0.956)	0.012	0.911 (0.774–1.073)	0.265	0.912 (0.788–1.055)	0.214
Active	0.522 (0.455–0.600)	<0.001	0.651 (0.565–0.749)	<0.001	0.685 (0.601–0.781)	<0.001
LTPA						
Inactive	1 (reference)		1 (reference)		1 (reference)	
Insufficiently active	0.741 (0.644–0.853)	<0.001	0.826 (0.714–0.956)	0.010	0.845 (0.728–0.980)	0.026
Active	0.506 (0.440–0.581)	<0.001	0.620 (0.537–0.716)	<0.001	0.652 (0.563–0.755)	<0.001
OPA						
Inactive	1 (reference)		1 (reference)		1 (reference)	
Insufficiently active	0.920 (0.720–1.175)	0.504	1.041 (0.808–1.340)	0.758	1.007 (0.779–1.301)	0.959
Active	0.780 (0.649–0.937)	0.008	0.925 (0.765–1.119)	0.424	0.910 (0.750–1.105)	0.342

OR, odds ratio; CI, confidence interval; TPA, total physical activity; LTPA, leisure time physical activity; OPA, occupational physical activity. Model 1: Unadjusted. Model 2: Adjusted for age Model 3: Model 2 + education, income, marital status, employment status, region, alcohol consumption, perceived stress level, energy intake, smoking status, occupational physical activity time (for leisure PA), leisure physical activity time (for occupational PA).

Analysis of the association between LTPA and MetS revealed that the insufficiently active (OR = 0.741, *p* < 0.001) and the active (OR = 0.506, *p* < 0.001) groups had a significantly lower OR for MetS than the inactive group, and the results remained significant in the age-adjusted model (model 2; insufficiently active, OR = 0.825, *p* = 0.010; Active, OR = 0.620, *p* < 0.001) and age-, SES-, and health-adjusted model (model 3; insufficiently active, OR = 0.845, *p* = 0.026; active, OR = 0.652, *p* < 0.001). Meanwhile, the active group (OR = 0.780, *p* = 0.008) had a significantly lower OR for OPA and MetS than the inactive group; however, the results were not significant in the age-adjusted model (OR = 0.925, *p* = 0.424) and age-, SES-, and health-adjusted model (OR = 0.910, *p* = 0.342).

### 3.5. Binomial logistic regression for the associations of smoking and PA with MetS

Table 5 presents the OR estimates for MetS based on PA level with respect to domain (TPA, LTPA, and OPA) and smoking status. The active smoking group (TPA, OR = 0.540, *p* < 0.001; LTPA, OR = 0.492, *p* < 0.001) showed a significantly lower OR for the association of TPA and LTPA with MetS than the inactive smoking group (OR = 1), and the results retained significance after adjusting for age, SES, and health-related mediators (TPA, OR = 0.679, *p* < 0.001; and LPA, OR = 0.624, *p* < 0.001). Non-smokers had a significantly lower OR for MetS than inactive smokers, irrespective of their TPA and LTPA duration, and the results were significant even after adjusting for all covariates. Meanwhile, no significant difference was found between the insufficiently active and inactive smoking groups.

**Table 5:** OR and 95% CI of metabolic syndrome according to physical activity and smoking status.

Variable	Model 1		Model 2		Model 3	
	OR (95% CI)	*p*-value	OR (95% CI)	*p*-value	OR (95% CI)	*p*-value
TPA						
Smoker						
Inactive	1 (reference)		1 (reference)		1 (reference)	
Insufficiently active	0.889 (0.745–1.060)	0.190	0.990 (0.825–1.188)	0.915	0.975 (0.811–1.172)	0.785
Active	0.540 (0.461–0.633)	<0.001	0.679 (0.576–0.801)	<0.001	0.679 (0.575–0.801)	<0.001
Non-smoker						
Inactive	0.589 (0.512–0.678)	<0.001	0.727 (0.629–0.842)	<0.001	0.781 (0.673–0.908)	0.001
Insufficiently active	0.450 (0.365–0.556)	<0.001	0.601 (0.483–0.747)	<0.001	0.642 (0.514–0.803)	<0.001
Active	0.354 (0.295–0.426)	<0.001	0.496 (0.409–0.600)	<0.001	0.540 (0.444–0.657)	<0.001
LTPA						
Smokers						
Inactive	1 (reference)		1 (reference)		1 (reference)	
Insufficiently active	0.847 (0.706–1.016)	0.073	0.937 (0.777–1.130)	0.497	0.928 (0.768–1.122)	0.438
Active	0.496 (0.412–0.597)	<0.001	0.620 (0.511–0.752)	<0.001	0.624 (0.513–0.758)	<0.001
Non-smokers						
Inactive	0.600 (0.526–0.685)	<0.001	0.736 (0.641–0.844)	<0.001	0.789 (0.685–0.910)	0.001
Insufficiently active	0.419 (0.338–0.520)	<0.001	0.545 (0.436–0.681)	<0.001	0.580 (0.462–0.729)	<0.001
Active	0.360 (0.296–0.439)	<0.001	0.494 (0.403–0.606)	<0.001	0.538 (0.436–0.664)	<0.001
OPA						
Smokers						
Inactive	1 (reference)		1 (reference)		1 (reference)	
Insufficiently active	0.961 (0.713–1.294)	0.791	1.056 (0.777–1.435)	0.727	1.029 (0.755–1.402)	0.857
Active	0.743 (0.596–0.926)	0.008	0.897 (0.715–1.126)	0.350	0.908 (0.721–1.143)	0.413
Non-smokers						
Inactive	0.569 (0.509–0.637)	<0.001	0.695 (0.618–0.781)	<0.001	0.765 (0.677–0.865)	<0.001
Insufficiently active	0.445 (0.286–0.693)	<0.001	0.649 (0.411–1.023)	0.063	0.735 (0.464–1.162)	0.188
Active	0.430 (0.307–0.601)	<0.001	0.613 (0.434–0.867)	0.006	0.701 (0.493–0.996)	0.047

OR, odds ratio; CI, confidence interval; TPA, total physical activity; LTPA, leisure time physical activity; OPA, occupational physical activity. Model 1: Unadjusted. Model 2: Adjusted for age. Model 3: Model 2 + education, income, marital status, employment status, region, alcohol consumption, perceived stress level, energy intake, occupational physical activity time (for leisure PA), and leisure physical activity time (for occupational PA).

Concerning the associations of smoking and OPA level with MetS, the active and non-smoker groups had a significantly lower OR for MetS than the inactive smoking group. Although this association sustained its significance even after adjusting for age, SES, and health-related mediators in the inactive and active non-smoking groups, the association did not retain significance in the active smoking and insufficiently active non-smoking groups after adjusting for covariates.

## 4. Discussion

This population-based cross-sectional study investigated the associations of smoking and PA levels in different types of PA with MetS in 7,229 adult men aged 19–64 years in Korea. The results showed that abstinence from smoking and moderate- to high-intensity TPA and LTPA of at least 150 min per week were associated with a lower risk of MetS. A novel finding of this study is that the risk of MetS decreases even among smokers if they engage in at least 150 min of moderate- to high-intensity TPA and LTPA.

Smoking directly decreases insulin sensitivity by activating the sympathetic nervous system and raising the circulating levels of cortisol, growth hormone, and free fatty acids, which accelerates visceral fat deposition (39). Additionally, the influx of smoking metabolites, such as nicotine and carbon monoxide, into the body contributes to increased insulin resistance (40) and unfavorable changes in the blood lipid profile (15). Thus, smoking has been recognized as a causative factor for MetS (40). Several epidemiological studies have consistently documented the increased risk of MetS in smokers, irrespective of potential covariates, such as race, sex, and SES (23, 31–43). This study found that non-smokers had a significantly lower risk of MetS than smokers, which was consistent with the findings of previous studies.

Furthermore, this study found an association between MetS and moderate- and high-intensity LTPA, which was also consistent with the findings of previous studies. A Spanish study investigating older adults reported that engaging in at least 150 min of moderate-intensity or 75 min of high-intensity LTPA per week was associated with a lower risk of MetS (44). A longitudinal study on adults with impaired glucose tolerance conducted in Finland also showed that increased participation in moderate- and high-intensity LTPA decreased the risk of MetS over an average follow-up period of 4.1 years (45). Moreover, a meta-analysis that included 17 longitudinal studies revealed that the risk of MetS declined with an increase in the duration of moderate- and high-intensity LTPA (46).

One major finding of this study is that engaging in at least 150 min of moderate- and high-intensity TPA and LTPA per week may offset the risk of MetS posed by smoking. This study is the first to analyze the interactive effect of smoking and PA on MetS in adult men using a nationally representative Korean sample. Some previous studies have examined the interactive impact of smoking and PA on MetS, albeit not extensively. For example, Huang et al. discovered that PA was associated with a normal lipid profile pertinent to MetS, irrespective of smoking status among workers of 20 companies in Taiwan (30), while Kim et al. showed that 8 weeks of PA decreased the WC in male college students in their 20s who smoked (31), highlighting the benefits of PA for MetS among smokers. However, these studies merely showed that engaging in PA is associated with improvement in some components of MetS without elucidating its association with MetS as a whole. Additionally, these studies were limited to workers and male college students in their 20s; therefore, the actual effects were probably undetected due to the relatively small sample size and lower statistical power. Moreover, these studies evaluated PA using a questionnaire or administered an exercise intervention and could not provide quantitative evidence supporting the benefits of PA, thereby limiting the generalizability of their findings. The current study showed that the risk of MetS decreased even among smokers when they performed at least 150 min of moderate- and high-intensity TPA and LTPA per week using a relatively large sample size, supporting and expanding on previous findings. Moreover, these findings highlight the importance of LTPA in minimizing the risk of smoking-related MetS and provide more detailed information about PA to help prevent the development of MetS among smokers.

A few theories have been proposed to explain the benefits of LTPA on MetS among smokers. First, the development of MetS is mediated by pro-inflammatory markers released by adipose tissue (47). In contrast to smoking, regular PA suppresses the production of pro-inflammatory markers, such as tumor necrosis factor-α, interleukin-6, and C-reactive protein (46, 47). This study’s findings suggest that the anti-inflammatory mechanism of regular PA lowers the risk of MetS by inhibiting the inflammatory response in the body caused by smoking. Second, regular PA enhances insulin sensitivity (48) and improves blood lipid profiles, irrespective of the increase in physical fitness or weight loss (49). Therefore, it is reasonable to infer that the benefits of PA on insulin sensitivity and blood lipids would offset the risk of higher FBG from smoking and its effects on blood lipid concentrations. Third, even smokers who regularly engage in LTPA are more likely to pursue a healthy lifestyle than those who do not engage in PA (50, 51), and the synergistic effect of a healthy lifestyle and LTPA may help alleviate the risk of MetS caused by smoking.

Meanwhile, these results indicated the absence of an association between OPA and MetS, regardless of smoking status. This is consistent with the PA paradox that OPA does not have health benefits. Increasing evidence suggests that although LTPA has various health benefits, including lowering the rate of cardiovascular disease and mortality, OPA has no health benefits but leads to unfavorable outcomes. Holtermann et al. conducted a longitudinal study on adult men and women living in Copenhagen, Denmark, and revealed that OPA was associated with the risk of mortality due to myocardial infarction, stroke, and other coronary artery diseases as well as the risk of all-cause mortality (52). Furthermore, Li et al. conducted a meta-analysis of 23 longitudinal studies including 790,000 participants and reported that moderate- and high-level OPA increased the risk of cardiovascular disease by 5–15% and 10–30%, respectively (53). The findings of this study show that OPA is unrelated to the improvement in MetS, which is consistent with those of previous studies.

The results on OPA can be interpreted as follows: First, long-term engagement in work involving muscle contractions elevates BP, and repeating this cycle may lead to chronic hypertension (54). Second, in contrast to LTPA, OPA is unintentional and is influenced by the nature of work and income level, and people with more physically demanding occupations generally have a lower SES (55). A low SES is linked to a high level of stress (56). High amounts of stress induce excessive production of glucocorticoids by the hypothalamic–pituitary–adrenal axis (57), which in turn inhibits insulin secretion and action (58), facilitates abdominal visceral fat deposition by stimulating adipocyte differentiation and proliferation, and lowers HDL-C levels by reducing the activity of lipoprotein lipases (59, 60). Ultimately, a high level of OPA is linked to stress, and the association between OPA and the MetS index may be mediated by stress. Previous epidemiological studies (61) reporting respective associations between OPA and stress and workplace stress and MetS (62, 63) support this explanation. Third, individuals with low levels of OPA generally devote a substantial amount of time to sedentary jobs (64). Sedentary time is an independent risk factor for MetS and bears a dose-dependent relationship with MetS prevalence (65). Therefore, essentially, people with low and high OPA both face a high risk of MetS, and the lack of association between OPA and MetS in this study can be explained based on these previous findings.

Although this study elucidates the benefits of LTPA for MetS, irrespective of smoking status, in a nationally representative sample of Korean adults, it has a few limitations. First, the cross-sectional study design precluded inference of causality. Thus, additional studies are needed to investigate the cellular and molecular mechanisms through which LTPA alleviates the risk of MetS caused by smoking. Second, this study’s findings may differ between sexes. However, in this study, women were excluded owing to the high discrepancy between the self-reported and actual smoking rates in women. Third, smoking and PA assessments primarily depended on self-reported data, which increases the risk of overestimation, underestimation, and recall bias. Therefore, the results should be interpreted cautiously, and longitudinal studies and research using objective instruments that can accurately assess the intensity and duration of PA in various domains are warranted. Despite these limitations, this study is based on a representative sample of systematically surveyed Korean adults. Additionally, this is the first study to report that LTPA can mitigate the risk of MetS due to smoking.

## 5. Conclusion

This study showed that engaging in moderate- and high-intensity LTPA can lower the risk of MetS among smokers, highlighting the clinical significance of moderate- and high-intensity LTPA interventions as a strategy to minimize the risk of MetS among smokers who find it difficult to quit the habit. However, exercise intervention studies applying various exercise types or follow-up studies of longitudinal design methods using objective tools to accurately evaluate the intensity and duration of PA in various areas are needed to better understand the mitigating effects of LTPA on the risk of MetS in smokers.

## Data availability statement

The datasets presented in this study can be found in online repositories. The names of the repository/repositories and accession number(s) can be found at: Korea National Health & Nutrition Examination Survey - from Korea Centers for Disease Control and Prevention agency. Accession Number: 2013-12EXP-03-5C, 2018-01-03-P-A, 2018-03-03-C-A, 2018-01-03-2C-A, 2018-03-03-5C-A. Repository URL: http://knhanes.kdca.go.kr/knhanes/sub03/syb03_02_05.do.

## Ethics statement

The studies involving humans were approved by Korea National Health and Nutrition Examination Survey (KNHANES) (2014–2021). The studies were conducted in accordance with the local legislation and institutional requirements. The participants provided their written informed consent to participate in this study.

## Author contributions

MK: Conceptualization, Data curation, Formal analysis, Investigation, Methodology, Writing – original draft, Writing – review & editing. JK: Data curation, Methodology, Writing – review & editing. IL: Conceptualization, Data curation, Formal analysis, Funding acquisition, Investigation, Methodology, Writing – original draft, Writing – review & editing.
